# Percutaneous Coronary Intervention before or after Transcatheter Aortic Valve Replacement: A Systematic Review and Meta-Analysis Involving 1531 Patients

**DOI:** 10.3390/jcm13123521

**Published:** 2024-06-16

**Authors:** Rodolfo Caminiti, Alfonso Ielasi, Giampaolo Vetta, Antonio Parlavecchio, Domenico Giovanni Della Rocca, Dario Pellegrini, Mariano Pellicano, Carolina Montonati, Nastasia Mancini, Gabriele Carciotto, Manuela Ajello, Giustina Iuvara, Francesco Costa, Giulia Laterra, Marco Barbanti, Fabrizio Ceresa, Francesco Patanè, Antonio Micari, Giampiero Vizzari

**Affiliations:** 1 U.O. Cardiologia Ospedaliera, IRCCS Ospedale Galeazzi-Sant’Ambrogio, 20157 Milan, Italy; rodolfocaminiti@msn.com (R.C.); alfonso.ielasi@gmail.com (A.I.); dar.pellegrini@gmail.com (D.P.); marianopellicano@libero.it (M.P.); carolina.montonati@gmail.com (C.M.); 2 Divisione di Cardiologia–Emodinamica, Policlinico Madonna della Consolazione, 89124 Reggio Calabria, Italy; 3 Heart Rhythm Management Centre, Universitair Ziekenhuis Brussel-Vrije Universiteit Brussel, European Reference Networks Guard-Heart, 1050 Brussels, Belgium; giampaolovetta7@gmail.com (G.V.); domenicodellarocca@hotmail.it (D.G.D.R.); 4 Interventional Cardiology Unit, Department of Clinical and Experimental Medicine, University of Messina, 98122 Messina, Italy; antonioparlavecchio1@gmail.com (A.P.); gcarciotto97@gmail.com (G.C.); manuela.ajello@outlook.it (M.A.); giustina.iuvara@gmail.com (G.I.); dottfrancescocosta@gmail.com (F.C.); micariantonio@gmail.com (A.M.); 5 Department of Clinical, Special and Dental Sciences, Marche Polytechnic University, 60131 Ancona, Italy; nastasiamancini92@gmail.com; 6 Faculty of Medicine and Surgery, Università degli Studi di Enna “Kore”, 94100 Enna, Italy; giulia.laterra1990@gmail.com (G.L.); mbarbanti83@gmail.com (M.B.); 7 Department of Cardiothoracic Surgery, Papardo Hospital, 98158 Messina, Italy; fabrizioceresa@aopaprdo.it (F.C.); f_patane@hotmail.com (F.P.)

**Keywords:** transcatheter aortic valve implantation, coronary artery disease, PCI, aortic stenosis

## Abstract

**Background:** The optimal timing to perform percutaneous coronary interventions (PCIs) in patients undergoing transcatheter aortic valve replacement (TAVR) is not well established. In this meta-analysis, we aimed to compare the outcomes of patients undergoing PCI before versus after TAVR. **Methods:** A comprehensive literature search was performed including Medline, Embase, and Cochrane electronic databases up to 5 April 2024 for studies that compared PCI before and after TAVR reporting at least one clinical outcome of interest (PROSPERO ID: CRD42023470417). The analyzed outcomes were mortality, stroke, and myocardial infarction (MI) at follow-up. **Results:** A total of 3 studies involving 1531 patients (pre-TAVR PCI *n* = 1240; post-TAVR PCI *n* = 291) were included in this meta-analysis following our inclusion criteria. Mortality was higher in the pre-TAVR PCI group (OR: 2.48; 95% CI: 1.19–5.20; *p* = 0.02). No differences were found between PCI before and after TAVR for the risk of stroke (OR: 3.58; 95% CI: 0.70–18.15; *p* = 0.12) and MI (OR: 0.66; 95% CI: 0.30–1.42; *p* = 0.29). **Conclusions:** This meta-analysis showed in patients with stable CAD undergoing TAVR that PCI after TAVR is associated with lower mortality compared with PCI before TAVR.

## 1. Introduction

Transcatheter aortic valve replacement (TAVR) revolutionized the treatment of severe aortic valve stenosis (AS), providing a less invasive alternative to surgical aortic valve replacement (SAVR) for elderly patients in all surgical risk categories [1,2,3]. TAVR has become a preferred option due to its minimally invasive nature, shorter recovery time, and reduced perioperative risks compared to traditional aortic valve surgery. An important aspect of managing patients undergoing TAVR is the treatment of concomitant coronary artery disease (CAD), which is common in patients with severe AS. Studies reported that CAD is present in approximately 40% to 75% of these patients. The coexistence of CAD adds complexity to the management strategy, particularly with regard to the optimal timing of coronary revascularization [4].

Percutaneous coronary intervention (PCI) prior to TAVR has been traditionally encouraged to optimize coronary perfusion and minimize the risk of ischemic events during the hemodynamic stress associated with transcatheter heart valve (THV) implantation [5]. This approach is consistent with current guidelines, which recommend prioritizing PCI for coronary artery lesions with greater than 70% stenosis in proximal segments or greater than 50% stenosis in left main disease [6]. The rationale behind this recommendation is to ensure adequate myocardial perfusion, thereby reducing the potential for perioperative myocardial infarction and improving overall outcomes. However, recent research has begun to question the need for routine PCI prior to TAVR, suggesting a more selective and individualized approach. Some studies support delaying coronary revascularization, particularly in cases where coronary lesions are not hemodynamically significant or in non-critical segments. This change in strategy aims to minimize the potential risks associated with PCI, such as bleeding complications and the need for dual antiplatelet therapy (DAPT) during the periprocedural phase of TAVR. Reducing the duration or need for DAPT is particularly beneficial in elderly and frail patients, who are more prone to bleeding and other complications [7]. In addition, recent advances in imaging techniques and hemodynamic assessment tools have made it possible to better identify which coronary lesions effectively require intervention. This allows for a more targeted approach, treating only those lesions that pose a significant risk to the patient’s health. By avoiding unnecessary PCI, patients can benefit from reduced procedural risks, shorter hospital stays, and potentially improved overall outcomes.

While TAVR has significantly modified the treatment of severe AS, the management of concomitant CAD remains a critical aspect of patient care. The aim of this meta-analysis is to investigate the optimal timing of PCI in patients undergoing TAVR.

## 2. Materials and Methods

### 2.1. Data Sources and Searches

We systematically searched the Medline, Embase, and Scopus electronic databases for studies published until 5 April 2024, focusing on those comparing the efficacy and safety of PCI prior to or after TAVR and reporting at least one clinical outcome of interest. Two investigators (R.C. and G.V.) independently conducted searches and article screening using the following terms: “transcatheter aortic valve implantation” and “percutaneous coronary intervention”. Disagreements were resolved by consensus. Detailed information on our literature search strategy is available in the Supplementary Materials in the Expanded Methods.

### 2.2. Study Selection

The Preferred Reporting Items for Systematic Reviews and Meta-Analyses (PRISMA) statement for reporting systematic reviews and meta-analyses was employed in this study to report the systematic review and meta-analysis. The predefined protocol was registered to the international prospective registry of systematic reviews (POSPERO ID: CRD42024535699). Two reviewers (R.C. and G.V.) independently performed the full-text selection, and disagreements were resolved by consensus. Studies had to meet the following criteria in order to be included in the analysis: (1) adult (≥18 years) population; (2) direct comparison between PCI before and after TAVR, (3) ≥6 months clinical follow-up available; and (4) one or more clinical outcomes of interest reported (e.g., stroke, myocardial infarction, all-cause death). Case reports, editorials, reviews, expert opinions, and studies not published in the English language were excluded.

### 2.3. Data Extraction and Quality Appraisal

Two investigators (R.C. and G.V.) extracted data from each trial using standardized protocol and reporting forms. In case of differences in extracted data, two investigators reassessed the manuscript together. Two reviewers (R.C. and G.V.) independently assessed quality items and disagreements were resolved by consensus. The Newcastle–Ottawa Quality Assessment Scale for cohort studies was used by two investigators (R.C. and G.V.) to assess the quality of each study.

### 2.4. Study Endpoints

The endpoints investigated were mortality, myocardial infarction (MI), and stroke. All endpoints were commonly defined according to the Academic Research Consortium definitions, Valve Academic Research Consortium (VARC)-2 and VARC-3 criteria used in the studies [8].

### 2.5. Statistical Analysis

Descriptive statistics are presented as mean and standard deviation (SD) for continuous variables, or number of cases (*n*) and percentage (%) for dichotomous and categorical variables. If studies reported continuous variables as median (interquartile range), Wang et al.’s method was employed for conversion into estimated mean  ±  standard deviation [9]. The Mantel–Haenszel odds ratio (OR) model was used to summarize the data for binary outcomes between treatment arms. Adjusted hazard ratios for mortality were examined in each study separately and were pooled according to a random-effects model with generic inverse variance weighting, calculating the risk estimates with 95%. Heterogeneity between studies was assessed using the Chi^2^, Tau^2^, and Higgins-I^2^ statistics, and random-effects models by Restricted maximum likelihood were used. Publication bias was assessed using funnel plots. Statistical analysis was performed with Review Manager (RevMan) (computer program) version 5.4.1, Copenhagen, Denmark: Nordic Cochrane Centre, The Cochrane Collaboration, 2020.

## 3. Results

### 3.1. Study Selection and Baseline Characteristics

**Figure 1:**
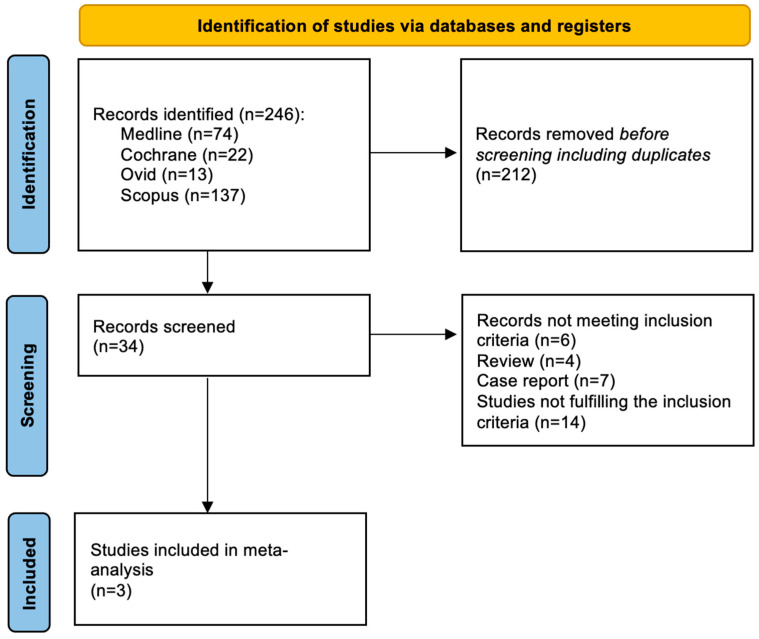
Evidence search and selection of Preferred Reporting Items for Systematic Reviews and Meta-Analyses (PRISMA).

Among 246 screened articles, 34 full texts were retrieved and reviewed for possible inclusion; a total of 3 studies fulfilled the selection criteria and were included in the final analysis (Figure 1).

The studies enrolled *n* = 1531 patients (pre-TAVR PCI group: *n* = 1240 patients; post-TAVR PCI group: *n* = 291 patients). Overall, 62.2% (95% CI: 59.6–62.8%) of patients were male with an average age of 80.5 years (95% CI: 80.1–81.2). A total of 62% (95% CI: 59.6–62.8%) of patients suffered from hypertension and 35.7% (95% CI: 35.2–36.3%) from diabetes. COPD was present in 21.1% (95% CI: 19.0–23.3%) of patients. The average LVEF was 56% (95% CI: 55.3–57.0%). Further details on baseline characteristics and clinical and angiographic follow-up times of the study population are reported in Table 1.

### 3.2. Endpoints

**Figure 2:**
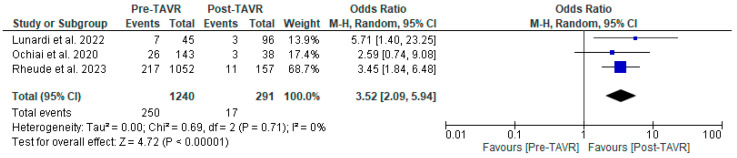
Forest plots comparing mortality between PCI pre- and post-TAVR. CI: confidence interval, M-H: Mantel–Haenszel, TAVR: transcatheter aortic valve replacement [10,11,12].

**Figure 3:**
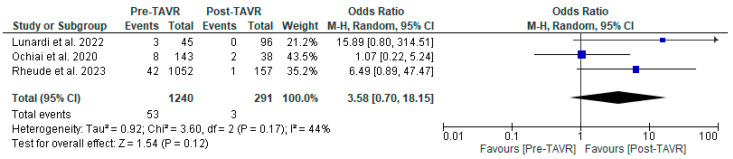
Forest plots comparing stroke events between PCI pre- and post-TAVR. CI: confidence interval, M-H: Mantel–Haenszel, TAVR: transcatheter aortic valve replacement [10,11,12].

**Figure 4:**
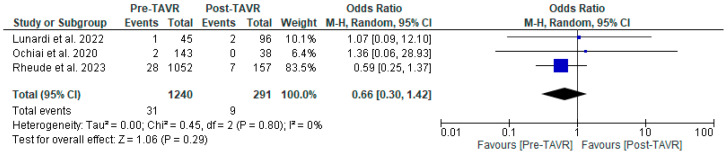
Forest plots comparing myocardial infarction between PCI pre- and post-TA VR. CI: confidence interval, M-H: Mantel–Haenszel, TAVR: transcatheter aortic valve replacement [10,11,12].

**Figure 5:**
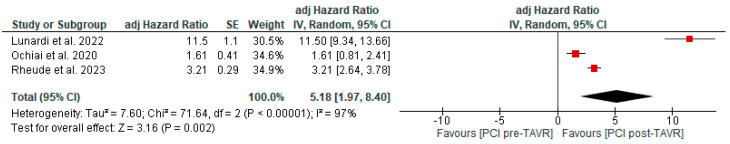
Adjusted hazard ratio comparing mortality between PCI pre- and post-TAVR. CI: confidence interval, M-H: Mantel–Haenszel, TAVR: transcatheter aortic valve replacement [10,11,12].

All studies reported clinical follow-up data on mortality, stroke, and myocardial infarction [10,11,12]. Mortality was significantly higher in the pre-TAVR PCI group [20% vs. 5.8%; RR: 3.52 (95% CI: 2.09–5.94; *p* < 0.001; I^2^ = 0%, *p* = 0.71] (Figure 2). No differences were found between PCI before and after TAVR for the risk of stroke [4.2% vs. 1.0%; RR: 3.58 (95% CI: 0.70–18.15; *p* = 0.12; I^2^ = 44%, *p* = 0.17] (Figure 3) and MI [2.5% vs. 3.0%; RR: 0.66 (95% CI: 0.30–1.42; *p* = 0.29; I^2^ = 0%, *p* = 0.80] (Figure 4). Adjusted HR for mortality was reported in all studies. Adjusted HR for mortality was significantly higher in the pre-TAVR PCI than in the post-TAVR PCI group (adjusted HR 5.18; 95% CI: 1.97–8.40; *p* = 0.002; I^2^ = 97%, *p* < 0.0001) (Figure 5).

### 3.3. Publication Bias

A graph and summary of the Newcastle–Ottawa Quality Assessment Scale for cohort studies is reported in Supplemental Figure S1. The funnel plots for visual inspection of the bias are reported in Supplemental Figure S2.

## 4. Discussion

TAVR has become a revolutionary treatment for severe AS. However, a significant proportion of these patients also have concomitant CAD, requiring a strategic approach to the effective management of both conditions [4]. PCI is often required in these patients, but the optimal timing of PCI in relation to TAVR remains the subject of ongoing research and debate. Our meta-analysis aimed to investigate the optimal timing of PCI in patients with significant CAD undergoing TAVR.

The main findings of our meta-analysis are as follows:

- Post-TAVR PCI lowers all-cause mortality: PCI performed after TAVR was associated with a significantly lower all-cause mortality rate compared to PCI performed before TAVR.
- Comparable myocardial infarction and stroke rates: the rates of myocardial infarction and stroke were almost comparable between the groups that received PCI before and after TAVR.

A staged PCI strategy prior to TAVR has been commonly adopted in recent years. This approach is based on the premise that treating significant coronary stenoses before valve replacement can help ensure optimal myocardial perfusion during the hemodynamic stress of TAVR. Conversely, high-risk CAD in the presence of severe AS needs to be treated cautiously to prevent ischemic complications [5,13]. It is noted that certain CAD subsets, such as proximal left anterior descending artery stenosis and left main coronary artery disease, must be managed with particular attention as indicated in the current guidelines [6]. These lesions are critical due to their potential to significantly impact myocardial blood supply. Current guidelines emphasize the importance of addressing these high-risk lesions before TAVR to minimize the risk of adverse cardiac events.

The REVASC-TAVI registry (*n* = 1603) collected data from patients undergoing TAVR with concomitant CAD and planned PCI (before, during, or after the interventional procedure on the aortic valve). The results showed that concomitant PCI was associated with the highest rates of acute kidney injury (AKI) and in-hospital mortality, while PCI after TAVR was associated with significantly lower rates of all-cause mortality and a composite endpoint of all-cause death, myocardial infarction, and stroke [10]. The study by Ochiai et al. (*n* = 1756) showed no difference in mid-term outcomes (major adverse cardiac and cerebrovascular events defined as a composite of all-cause death, myocardial infarction, unplanned revascularization, and stroke) between PCI before, during, or after TAVR. Notably, patients in the PCI after TAVR group were only treated with a BE THV [11]. The results of Lunardi et al. (*n* = 1162) showed a higher risk of stroke in the PCI before TAVR group and almost comparable overall procedural and in-hospital adverse events between the two groups (before and after), regardless of trans-catheter heart valve type or CAD complexity. Furthermore, long-term clinical outcomes (cardiac death, target lesion failure, target vessel failure, target lesion revascularization, target vessel revascularization, stroke, or acute MI) support TAVR before PCI with favorable overall survival [12].

### 4.1. Coronary Physiology Assessment

Invasive assessment of intermediate stenosis by coronary physiology using indices such as fractional flow reserve (FFR) or derived indices such as instantaneous wave-free ratio (iFR) is challenging in patients with stable CAD and AS. Aortic stenosis may alter coronary physiology, making these indices less reliable. Indeed, patients with severe AS have systolic left ventricular (LV) outflow obstruction, elevated LV end-diastolic pressure, and significant LV hypertrophy [14,15]. These factors will preferentially reduce systolic over diastolic coronary blood flow, thereby influencing the invasive physiological measurement. iFR may be a better option than FFR in patients with AS because it does not require adenosine and is independent of systolic flow [16]. Therefore, despite some observational data showing no significant changes when performing physiological assessment before or after TAVR, PCI after TAVR may theoretically provide a more accurate assessment of coronary physiology and may be beneficial in determining the need for revascularization [17,18].

### 4.2. Renal Function and Contrast-Induced Nephropathy

Renal function is a critical consideration in patients undergoing both PCI and TAVR, particularly because of the risk of contrast-induced nephropathy (CIN). CIN is a form of acute kidney injury (AKI) caused by the iodinated contrast media used in these procedures. Patients with pre-existing renal impairment are at increased risk of CIN, which can lead to prolonged hospitalization, increased morbidity, and even mortality. The incidence of CIN is a major concern as it can complicate both PCI and TAVR procedures. Performing PCI prior to TAVR can expose patients to a higher cumulative dose of contrast, increasing the risk of AKI [12]. This risk is exacerbated in elderly patients, who often have impaired renal function and multiple comorbidities. Strategies to mitigate this risk include using the minimum necessary contrast volume, using ultra-low or zero-contrast techniques, and ensuring adequate hydration and renal protection before, during, and after the procedure [19]. In addition, delaying PCI after TAVR may reduce cumulative contrast exposure and allow for better preservation of renal function, making it a potentially safer approach for treating patients with concomitant CAD and severe AR.

### 4.3. Technical Considerations

The choice of THV type plays an important role in the decision to perform PCI before or after TAVR. Balloon-expandable (BE) THVs with short frame heights facilitate coronary re-access compared to self-expanding (SE) THVs with tall frames and supra-annular leaflet positions. Recent advances in techniques and devices to precisely align the THV posts with the native aortic valve commissures have further reduced the challenges associated with SE THVs and support the feasibility of performing PCI after TAVR [20,21,22]. However, prior PCI must be considered in TAVR-in-SAVR or TAVR-in-TAVR procedures. In these scenarios, coronary access can be even more challenging: in up to half of the cases, changes in sinus geometry, pushing aside of the leaflets of the first THV by the second, and misalignment of the commissures may contribute to impaired coronary engagement [23].

### 4.4. Dual Antiplatelet Therapy

Dual antiplatelet therapy (DAPT) is a key component in the management of patients undergoing TAVR and percutaneous coronary intervention (PCI). DAPT, typically consisting of aspirin and a P2Y12 inhibitor, is essential to prevent thrombotic complications following stent implantation in PCI and to minimize the risk of valve thrombosis following TAVR [24]. However, the timing of PCI relative to TAVR adds complexity to DAPT management. When PCI is performed before TAVR, patients require immediate DAPT to maintain stent patency, which may increase the risk of bleeding complications during the subsequent TAVR procedure. This is of particular concern in elderly and frail patients, who are already at higher risk of bleeding. Conversely, delaying PCI after TAVR allows the valve procedure to be completed with potentially less aggressive antithrombotic therapy, thereby reducing the immediate peri-procedural bleeding risk [7]. However, this approach requires careful post-TAVR management to balance the need for DAPT for stent protection against the increased risk of bleeding, particularly in those who may also require anticoagulation for other conditions such as atrial fibrillation [25].

### 4.5. Evidence from Randomized Trials and Future Research

Although most of the studies included in our meta-analysis are observational and retrospective, our results are consistent with the only randomized study available: the ACTIVATION trial (PercutAneous Coronary in Tervention prIor to transcatheter aortic VAlve implanta TION). This trial evaluated adverse outcomes in 235 patients undergoing TAVR with significant CAD who were randomized to receive PCI or no PCI prior to aortic valve replacement. At 1-year follow-up, the trial reported equivalent outcomes in terms of death and rehospitalization with a higher rate of bleeding in PCI before TAVR [13]. Further prospective studies are expected to provide robust evidence and guidance on the correct timing of PCI in patients undergoing TAVR. The TAVI-PCI trial (ClinicalTrials.gov: NCT04310046) (*n* = 900) will randomize patients to PCI 1–45 days before or after TAVR with a BE THV guided by FFR. The aim of this study is to evaluate the value of invasive assessment of coronary physiology in patients with AS and to investigate the performance of iFR. The COMPLETE TAVR trial (ClinicalTrials.gov: NCT04634240) is currently randomizing patients undergoing complete revascularization to medical therapy after TAVR with a large study population of 4000 patients with severe CAD (stenosis in a segment greater than 2.5 mm in diameter by visual angiographic assessment).

In conclusion, our meta-analysis supports performing PCI after TAVR; however, it is important to consider patient-specific factors that may influence the timing of PCI. These factors include age, LVEF, extent and severity of CAD, and institutional expertise. A personalized approach, based on a careful assessment of individual patient factors and shared decision-making between patients and healthcare providers, is essential to provide optimal, tailored therapy for each patient.

### 4.6. Limitations

This meta-analysis includes several limitations. We were able to include only a small number of observational studies, thus leading to a high risk of bias in the results. To overcome the presence of possible confounding factors, we performed a pooled analysis of adjusted HR, which confirmed that pre-TAVR PCI was associated with an increased risk of mortality at follow-up compared to post-TAVR PCI. However, the result for adjusted HR was affected by high heterogeneity. The length of follow-up in the included trials was not standardized and was mostly based on the discretion of the operators and the clinical condition of the patients. The funnel plot for visual inspection of the bias cannot be correctly interpreted with such a small number of studies.

## 5. Conclusions

In patients with concomitant severe aortic valve stenosis and significant CAD undergoing TAVR, a strategy of postponing PCI is associated with lower all-cause death at follow-up while similar MI and stroke rates as compared to a pre-TAVR PCI. Further randomized trials are awaited to confirm our findings.

## Figures and Tables

**Table 1:** Main characteristics of studies included in the meta-analysis.

	PCI Timing	Rheude et al. [10]	Ochiai et al. [11]	Lunardi et al. [12]
Study year		2023	2020	2022
Follow-up		720 days	603 (interquartile range: 346 to 1017) days	15 months (interquartile range: 5–28 months)
Patients, *n*	Pre TAVR	1052	143	45
	Post TAVR	157	38	96
Age, years	Pre TAVR	82.2 (78.5–85.3)	82.4 ± 7.8	80.45 ± 7.23
	Post TAVR	82.0 (79.0–85.2)	78.9 ± 10.6	81.89 ± 6.01
Female, *n*	Pre TAVR	430	42	18
	Post TAVR	66	8	42
STS score, %	Pre TAVR	5.0 (3.2–5.0)	5.3 (3.5−7.8)	5.45 ± 5.25
	Post TAVR	5.0 (3.3–5.1)	4.7 (2.8−6.3)	4.12 ± 3.67
Hypertension, *n*	Pre TAVR	892	129	39
	Post TAVR	135	37	85
DM, *n*	Pre TAVR	331	47	16
	Post TAVR	52	12	31
Prev. CABG, *n*	Pre TAVR	89	32	7
	Post TAVR	9	8	7
Prev. MI, *n*	Pre TAVR	208	35	12
	Post TAVR	27	6	20
COPD, *n*	Pre TAVR	154	31	12
	Post TAVR	24	7	13
AF, *n*	Pre TAVR	289	22	9
	Post TAVR	34	10	24
EF, %	Pre TAVR	58.0 (48.0–63.0)	53.2 ± 15.6	48.65 ± 12.76
	Post TAVR	59.5 (48.0–60.0)	51.1 ± 14.3	54.87 ± 12.88

AF: atrial fibrillation, EF: ejection fraction, CABG: coronary artery bypass graft, COPD: chronic obstructive pulmonary disease, DM: diabetes mellitus, MI: myocardial infarction, STS: society of thoracic surgeons, TAVR: transcatheter aortic valve replacement. Values are expressed as mean and ± SD; median and IQR (min–max).

## Data Availability

The raw data supporting the conclusions of this article will be made available by the authors upon request.

## Supplementary Materials

The following supporting information can be downloaded at: https://www.mdpi.com/article/10.3390/jcm13123521/s1, Figure S1: (A) Methodological quality graph and (B) methodological quality summary for the risk of bias from the included studies using the Newcastle–Ottawa Quality and Assessment Scale for Cohort Studies tool; Figure S2: Funnel plot for the mortality (A), stroke (B), myocardial infarction (C) and adjusted HR for mortality; Table S1: Study type, outcomes definition and Adjusted Hazard Ratios Covariates.

## References

[1] Jørgensen T.H., Thyregod H.G.H., Ihlemann N., Nissen H., Petursson P., Kjeldsen B.J., Steinbrüchel D.A., Olsen P.S., Søn-dergaard L. (2021). Eight-Year Outcomes for Patients with Aortic Valve Stenosis at Low Surgical Risk Randomized to Transcatheter Vs. Surg. Aortic Valve Replacement. Eur. Heart J..

[2] Forrest J.K., Deeb G.M., Yakubov S.J., Gada H., Mumtaz M.A., Ramlawi B., Bajwa T., Teirstein P.S., DeFrain M., Mup-pala M. (2023). 3-Year Outcomes After Transcatheter or Surgical Aortic Valve Replacement in Low-Risk Patients with Aortic Ste-Nosis. J. Am. Coll. Cardiol..

[3] Grüntzig A.R., Senning A., Siegenthaler W.E. (1979). Nonoperative Dilatation of Coronary-Artery Stenosis: Percutaneous Trans-luminal Coronary Angioplasty. N. Engl. J. Med..

[4] Danson E., Hansen P., Sen S., Davies J., Meredith I., Bhindi R.A. (2016). Treatment, and Prognostic Implications of CAD in Patients Undergoing TAVI. Nat. Rev. Cardiol..

[5] Wenaweser P., Pilgrim T., Guerios E., Stortecky S., Huber C., Khattab A.A., Kadner A., Buellesfeld L., Gloekler S., Meier B. (2011). Impact of Coronary Artery Disease and Percutaneous Coronary Intervention on Outcomes in Patients with Severe Aortic Stenosis Undergoing Transcatheter Aortic Valve Implantation. EuroIntervention.

[6] Vahanian A., Beyersdorf F., Praz F., Milojevic M., Baldus S., Bauersachs J., Capodanno D., Conradi L., Bonis M., Paulis R. (2021). ESC/EACTS Guidelines for the Management of Valvular Heart Disease: Developed by the Task Force for the Management of Valvular Heart Disease of the European Society of Cardiology (ESC) and the European Association for Car-Dio-Thoracic Surgery (EACTS). Eur. Heart J..

[7] Garot P., Neylon A., Morice M.-C., Tamburino C., Bleiziffer S., Thiele H., Scholtz S., Schramm R., Cockburn J., Cunning-ton M. (2022). Bleeding Risk Differences after TAVR According to the ARC-HBR Criteria: Insights from SCOPE 2. EuroIntervention.

[8] Kappetein A.P., Head S.J., Généreux P., Piazza N., van Mieghem N.M., Blackstone E.H., Brott T.G., Cohen D.J., Cutlip D.E., van Es G.-A. (2012). Updated Standardized Endpoint Definitions for Transcatheter Aortic Valve Implantation: The Valve Academic Research Consortium-2 Consensus Document (VARC-2). Eur. J. Cardio-Thoracic Surg..

[9] Wan X., Wang W., Liu J., Tong T. (2014). Estimating the Sample Mean and Standard Deviation from the Sample Size, Median, Range and/or Interquartile Range. BMC Med Res. Methodol..

[10] Rheude T., Costa G., Ribichini F.L., Pilgrim T., Amat Santos I.J., Backer O., Kim W.-K., Ribeiro H.B., Saia F., Bunc M. (2023). Comparison of Different Percutaneous Revascularisation Timing Strategies in Patients Undergoing Transcatheter Aortic Valve Implantation. EuroIntervention.

[11] Ochiai T., Yoon S.-H., Flint N., Sharma R., Chakravarty T., Kaewkes D., Patel V., Nakamura M., Cheng W., Makkar R. (2020). Timing and Outcomes of Percutaneous Coronary Intervention in Patients Who Underwent Transcatheter Aortic Valve Implanta-Tion. Am. J. Cardiol..

[12] Lunardi M., Venturi G., Del Sole P.A., Ruzzarin A., Mainardi A., Pighi M., Pesarini G., Scarsini R., Tavella D., Gottin L. (2022). Optimal Timing for Percutaneous Coronary Intervention in Patients Undergoing Transcatheter Aortic Valve Implantation. Int. J. Cardiol..

[13] Patterson T., Clayton T., Dodd M., Khawaja Z., Morice M.C., Wilson K., Kim W.-K., Meneveau N., Hambrecht R., Byrne J. (2021). ACTIVATION (PercutAneous Coronary inTervention prIor to Transcatheter Aortic VAlve implantaTION. JACC Cardiovasc. Interv..

[14] Zelis J.M., Tonino P.A.L., Pijls N.H.J., De Bruyne B., Kirkeeide R.L., Gould K.L., Johnson N.P. (2020). Coronary Microcirculation in Aortic Stenosis: Pathophysiology, Invasive Assessment, and Future Directions. J. Interv. Cardiol..

[15] Pesarini G., Scarsini R., Zivelonghi C., Piccoli A., Gambaro A., Gottin L., Rossi A., Ferrero V., Vassanelli C., Ribichini F. (2016). Functional Assessment of Coronary Artery Disease in Patients Undergoing Transcatheter Aortic Valve Implantation. Circ. Cardiovasc. Interv..

[16] Patel K.P., Michail M., Treibel T.A., Rathod K., Jones D.A., Ozkor M., Kennon S., Forrest J.K., Mathur A., Mullen M.J. (2021). Coronary Revascularization in Patients Undergoing Aortic Valve Replacement for Severe Aortic Stenosis. JACC Cardiovasc. Interv..

[17] Sabbah M., Olsen N.T., Holmvang L., Tilsted H.H., Pedersen F., Joshi F.R., Sørensen R., Jabbari R., Arslani K., Sondergaard L. (2023). Long-Term Changes in Coronary Physiology after Aortic Valve Replacement. EuroIntervention.

[18] Ahmad Y., Vendrik J., Eftekhari A., Howard J.P., Cook C., Rajkumar C., Malik I., Mikhail G., Ruparelia N., Hadjiloizou N. (2019). Determining the Predominant Lesion in Patients with Severe Aortic Stenosis and Coronary Stenoses: A Multicenter Study Using Intracoronary Pressure and Flow. Circ. Cardiovasc. Interv..

[19] Ielasi A., Buono A., Briguglia D., Uccello G., Pellicano M., Medda M., Tespili M. (2023). Minimalistic Contrast-Zero Aortic Valve-in-Valve Replacement Using the Novel Hydra Transcatheter Valve in a Patient with Severe Chronic Kidney Disease. Cardiovasc. Revasc. Med..

[20] Ielasi A., Caminiti R., Pellegrini D., Montonati C., Pellicano M., Giannini F., Briguglia D., De B.G., Guagliumi G., Tespili M. (2024). Technical Aspects for Transcatheter Aortic Valve Replacement with the Novel Balloon-Expandable Myval Octacor. JACC Cardiovasc. Interv..

[21] Barbanti M., Costa G., Picci A., Criscione E., Reddavid C., Valvo R., Todaro D., Deste W., Condorelli A., Scalia M. (2020). Coronary Cannulation after Transcatheter Aortic Valve Replacement. JACC Cardiovasc. Interv..

[22] Khera S., Krishnamoorthy P., Sharma S.K., Kini A.S., Dangas G.D., Goel S., Lerakis S., Anastasius M., Moreno P., Tang G.H.L. (2023). Improved Commissural Alignment in TAVR with the Newest Evolut FX Self-Expanding Supra-Annular Valve: First-in-Human Experience. JACC Cardiovasc Interv.

[23] Ochiai T., Chakravarty T., Yoon S.-H., Kaewkes D., Flint N., Patel V., Mahani S., Tiwana R., Sekhon N., Nakamura M. (2020). Coronary Access after TAVR. JACC Cardiovasc. Interv..

[24] Brouwer J., Nijenhuis V.J., Delewi R., Hermanides R.S., Holvoet W., Dubois C.L., Frambach P., De Bruyne B., van Houwelingen G.K., Van Der Heyden J.A. (2020). Aspirin with or without Clopidogrel after Transcatheter Aortic-Valve Implantation. N. Engl. J. Med..

[25] Hindricks G., Potpara T., Dagres N., Arbelo E., Bax J.J., Blomström-Lundqvist C., Boriani G., Castella M., Dan G.-A., Dilaveris P.E. (2021). 2020 ESC Guidelines for the Diagnosis and Management of Atrial Fibrillation Developed in Collaboration with the European Association for Cardio-Thoracic Surgery (EACTS): The Task Force for the Diagnosis and Management of Atrial Fibrillation of the European Society of Cardiology (ESC) Developed with the Special Contribution of the European Heart Rhythm Association (EHRA) of the ESC. Eur. Heart J..

