# Supplementary material for: Percutaneous Coronary Intervention before or after Transcatheter Aortic Valve Replacement: A Systematic Review and Meta-Analysis Involving 1531 Patients
Source: J Clin Med. 2024 Jun 16;13(12):3521. doi: 10.3390/jcm13123521 (PMC11204616; doi:10.3390/jcm13123521)
Supplement: Supplementary file 1 [file jcm-13-03521-s001.zip › jcm-3001241-supplementary.pdf]

SUPPLEMENTAL MATERIALS

Percutaneous coronary intervention before or after transcatheter aortic valve implantation: A Systematic Review and Meta-Analysis Involving 6531 Patients

Expanded Methods

Data Sources and Searches

Supplemental Figures

Supplemental Figure 1

Supplemental Figure 2

Supplemental Tables

Supplemental Table 1

Expanded Methods

Data Sources and Searches

We used the following search strings for Pubmed (1), Embase (2), Scopus (3):

1) ((Transcatheter aortic valve implantation) AND (percutaneous coronary intervention)) OR (TAVI) OR (PCI) OR (Myocardial Infarction)

2) Mesh descriptor: [Transcatheter aortic valve implantation] explode all trees OR percutaneous coronary intervention :ti OR TAVI and Myocardial Infarction:ti OR PCI

3) (percutaneous coronary intervention or PCI).ti. and (Transcatheter aortic valve implantation and Myocardial Infarction or MI or TAVI).ab.

Supplemental Figure 1: (A) Methodological quality graph and (B) methodological quality summary for the risk of bias from the included studies using the Newcastle–Ottawa Quality and Assessment Scale for Cohort Studies tool.

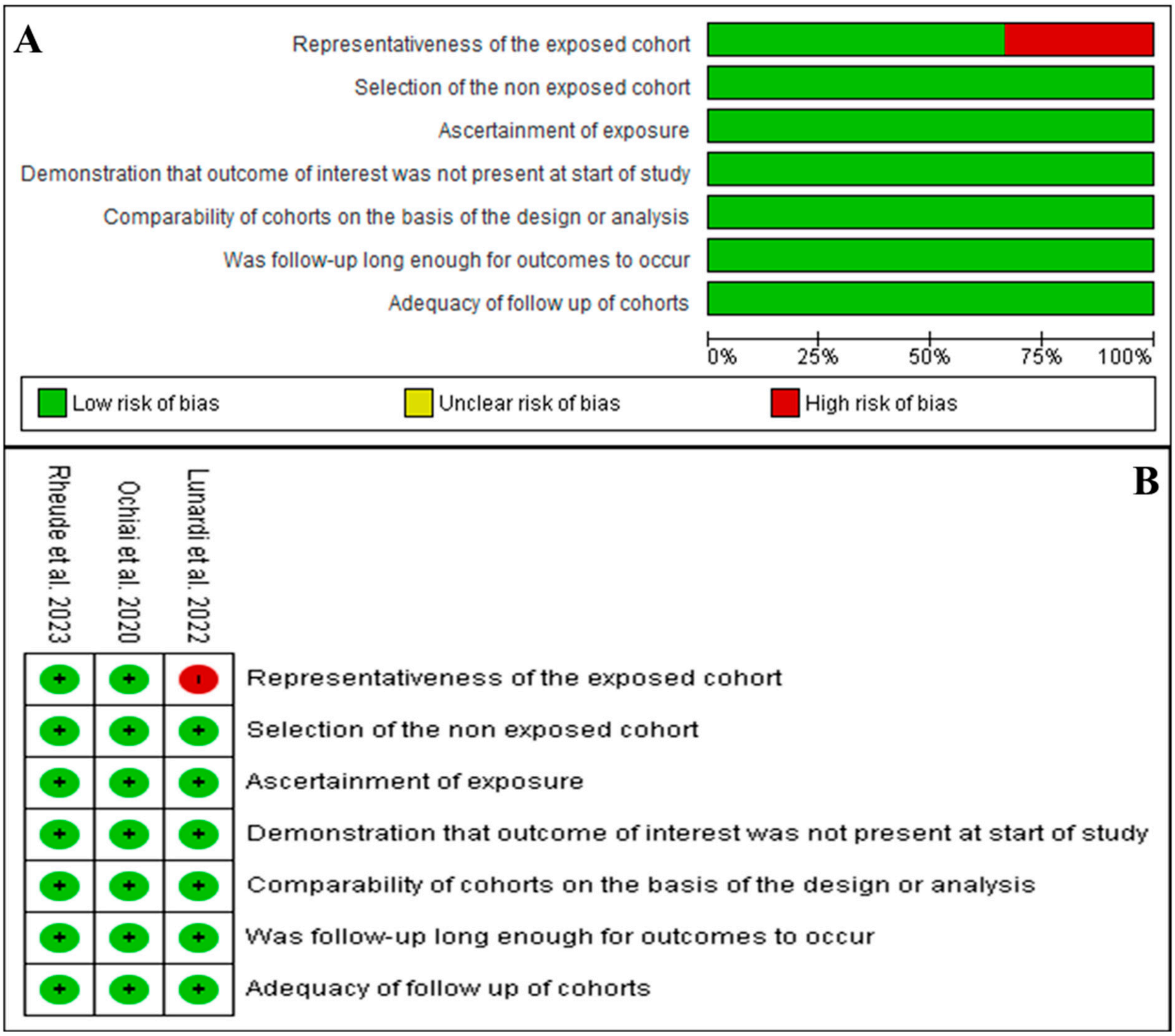

Supplemental Figure 2: Funnel plot for the mortality (A), stroke (B), myocardial infarction (C) and adjusted HR for mortality.

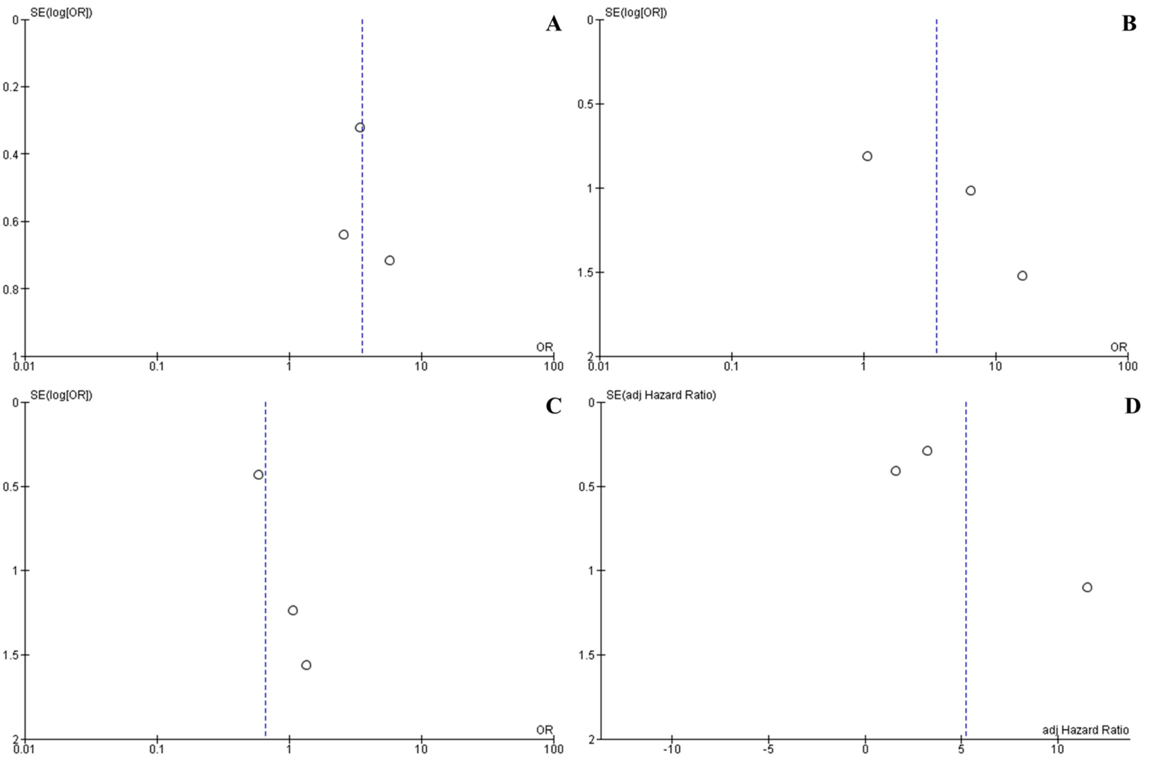

Supplemental Table 1: Study type, outcomes definition and Adjusted Hazard Ratios Covariates.

	Rheude et al	Ochiai et al	Lunardi et al
**Study Type**	retrospective, non-randomised study	retrospective observational study	retrospective analysis of prospectively collected data
**MACCE Definition**	All-cause death, stroke, MI or HF rehospitalisation	composite of all-cause death, myocardial infarction, unplanned revasculari-zation, and stroke.	occurrence of cardiac death, TLF, TVF, TLR, TVR, stroke or acute myocardial infarction.
**Adjusted Hazard Ratios Covariates**	age, sex, Society of Thoracic Surgeons score, Canadian Cardiovascular Society class, chronic obstructive pulmonary disease, diabetes mellitus, hypertension, New York Heart Association Class, atrial fibrillation, estimated glomerular filtration rate, prior pacemaker, prior stroke, prior coronary artery bypass graft, prior MI, left ventricular ejection fraction, mean transvalvular gradient, mul-tivessel CAD, LM or proximal left anterior descending artery CAD	male, previous coronary artery bypass grafting and ejection fraction	Glomerular filtration rate, LVEF, Sex, Age and PCI before TAVI

MI myocardial infarction, HF heart failure, TLF target lesion failure, TLR target lesione revascularization, TVR target vessel revascularization, LVEF left ventricle ejection fraction, LM left main
